# Multiscale Mechanical Responses of the Racetrack NbTi Superconducting Coil Under Dynamic Pressures

**DOI:** 10.3390/ma18174072

**Published:** 2025-08-30

**Authors:** Wei Liu, Lianchun Wang, Peng Ma, Yong Li, Wentao Zhang, Peichang Yu, Qiang Chen, Yongbin Wang, Weiwei Zhang

**Affiliations:** 1 Western Superconducting Technologies Co., Ltd., Xi’an 710014, China; mapeng@c-wst.com (P.M.); ctly@c-wst.com (Y.L.);; 2 Xi’an Superconducting Magnet Technologies Co., Ltd., Xi’an 710014, China; 3 College of Intelligence Science and Technology, National University of Defense Technology, Changsha 410000, China; yupeichang@nudt.edu.cn (P.Y.); chenqiang08@nudt.edu.cn (Q.C.); 4 School of Science, Lanzhou University of Technology, Lanzhou 730050, China; wangyb19@lut.edu.cn (Y.W.);

**Keywords:** NbTi superconducting coil, multiscale simulation, friction contact, dynamic pressure

## Abstract

Racetrack NbTi superconducting coil is a key component in Maglev train systems due to its excellent mechanical processing performance and lower construction cost. However, dynamic pressures during high-speed operations can influence contact pressures and cause internal filament damage, leading to critical current degradation and quench, which threaten the stable operation of the superconducting magnet. Considering that the NbTi coil has a typical hierarchical structure and comprises thousands of filaments, this study constructs an efficient multiscale framework combining the finite element method (FEM) and self-consistent clustering analysis (SCA) to study the multiscale responses of the NbTi coil. The mechanical responses of the two-scale racetrack coil under monotonic and periodic pressures are investigated, and the effects of the friction contacts between strands are also discussed. The study reveals that internal contacts significantly influence local contact pressures and microscopic stresses, and periodic loading leads to stress accumulation with cycle times. The proposed framework efficiently captures critical microscale responses and can be applied to other multiscale materials and structures.

## 1. Introduction

NbTi superconducting materials have been broadly utilized and explored in scientific and commercial applications, such as accelerator magnets, magnetic resonance imaging, and Maglev trains [1,2,3,4]. In a Maglev train, the racetrack NbTi coil has become the main component in the lower-temperature magnet system due to its excellent mechanical processing performance and lower construction cost [5]. Compared with conventional superconducting magnets, vehicle-mounted superconducting magnets operate in a dynamic, high-speed motion environment. The stability levels of the electromagnetic structure, mechanical structure, and low-temperature cooling system are restricted by many influencing factors [6].

One critical factor is the impact of the dynamic pressures from the train and surrounding air, which may hinder the stable operation of the superconducting coil; this should be considered in the design and operation of the magnet [7]. When the high-speed Maglev train enters a tunnel, the air pressure can change quickly and cause serious damage to the surrounding structures [7,8]. The racetrack NbTi coil should bear the dynamic pressures under such extreme conditions. The coil usually consists of many NbTi filaments, and damage to several internal filaments can decrease the critical current density and quench [9], leading to severe accidents and harming the stable operation of the train. Therefore, a comprehensive mechanical analysis of the racetrack NbTi coil is necessary. However, traditional numerical simulations usually utilize the sequential multiscale method, where the NbTi filaments and copper matrix are regarded as microscale homogenized composite materials, and the homogenized parameters are utilized in the macroscale simulation [10]. This method can reduce the computational complexity but at the cost of ignoring the mechanical responses of the microscale NbTi filaments and copper matrix. Multiscale simulation can address this problem by introducing the reduced-order method to solve the microscale computation and maintain the microscale responses [11,12]. Recently, coupling the finite element method (FEM) and reduced-order method to construct the multiscale framework has become popular, as in FEM-SCA (self-consistent clustering analysis) [13,14], FEM-FFT (fast Fourier transformation method) [15], FEM-ML (machine learning) [16,17], and so on. Since FEM-SCA can balance efficiency and precision, many researchers have utilized this method to simulate the service loading conditions for Nb_3_Sn coils and magnets [18,19], fiber-reinforced composites [20], polycrystalline materials [21], and so on. Considering the similar structures of the NbTi and Nb_3_Sn strands, we chose the FEM-SCA method to perform multiscale analysis of the racetrack NbTi coil.

Another important factor is the friction contact between the NbTi wires. In the assembly of the racetrack NbTi coil, each NbTi wire contains mainly NbTi filaments and copper matrix, and the friction contact between the NbTi wires can also be considered an important factor influencing the mechanical responses [22]. The vibration results indicate that the friction is caused by the relative microscopic sliding between the structural components of the superconducting coil [23]. When the coil is subjected to the dynamic pressure, the deformation and interaction with the outer steel coating can also affect the friction contact between the NbTi wires [24]. Therefore, we used a simple way of dividing the original NbTi wire array into small fractions, where each macroscopic element of the fraction represents a microscale representative volume element (RVE), and considered the friction contacts between the strands along different directions.

In this paper, we establish an efficient multiscale framework using FEM-SCA to analyze the multiscale mechanical responses of the racetrack NbTi superconducting coil under dynamic pressures. The SCA method is utilized to reduce the computational complexity of the microscale simulation, while the macroscopic structures of the NbTi coil are analyzed with FEM to obtain the overall responses. The remainder of this paper is organized as follows: in Section 2, the reduced-order SCA algorithm for microscale computation is briefly introduced and the FEM-SCA multiscale framework for the NbTi coil is constructed. In Section 3, the overall and local response comparisons of the microscale RVE obtained using SCA and FEM are illustrated to show the efficiency and precision of SCA. In Section 4, a two-scale analysis of the NbTi coil under monotone and periodic pressure is conducted, and the multiscale mechanical responses are investigated and compared at different loading times and positions. The effects of the friction contacts between different strands are also discussed. In Section 5, a summary of the paper is presented and research prospects are also proposed.

## 2. The Model and Methodology of Multiscale Analysis

**Figure 1:**
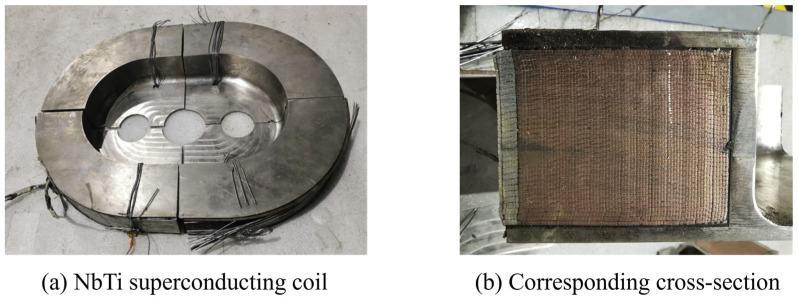
Sample of a racetrack NbTi superconducting coil (**a**) and its cross-section (**b**).

In this paper, we study the racetrack NbTi superconducting coil, which consists of two main components: the stainless steel coating and NbTi wires. To achieve high current density and stability, thousands of NbTi wires are often incorporated in the superconducting coil. Figure 1a,b present an actual NbTi superconducting coil and its corresponding cross-section, respectively. In Figure 1b, each small yellow rectangular region represents a single NbTi wire, and the outer silver rectangular region is the stainless steel coating. However, every NbTi wire is also a composite material that contains NbTi filaments and a copper matrix. Therefore, if we want to comprehensively model the racetrack NbTi superconducting coil, the hierarchical structure must be considered and multiscale analysis is necessary.

### 2.1. The Multiscale Geometry Model of the Racetrack NbTi Superconducting Coil

**Figure 2:**
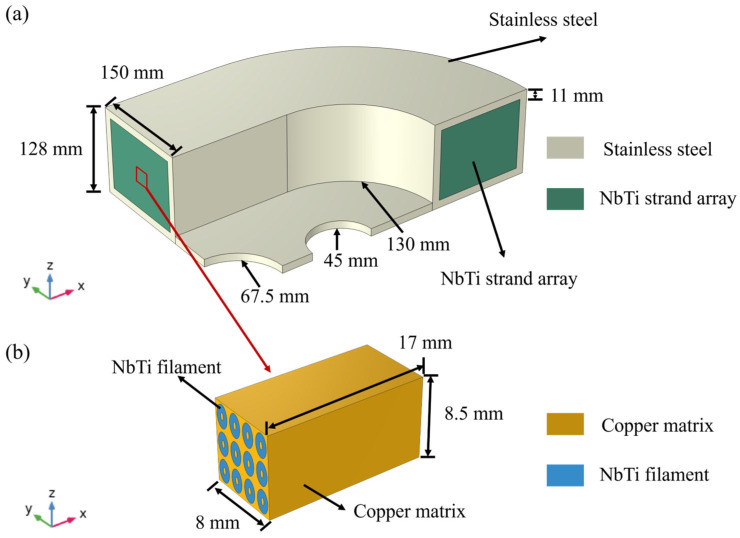
The macroscale (**a**) and microscale (**b**) geometry models of the racetrack NbTi superconducting coil.

To fully describe the geometric features of the racetrack NbTi superconducting coil, corresponding numerical models were established and are shown in Figure 2. The macroscale quarter model consists of the outer coating and the stainless steel and inner NbTi wire array connections between the two ends. The quarter model was adopted to reduce the computational degrees and meshes, and the corresponding geometric parameters are also depicted in Figure 2.

Each fraction of the NbTi wire array represents a microscale RVE model with NbTi filaments and copper matrix, as shown in Figure 2b. For simplicity, the insulation layer between strands is not considered, and the NbTi filament along the *x* direction is regarded as straight. The copper matrix occupies the inner and outer regions of the RVE, and the NbTi filaments are in the middle regions. As seen in Figure 1b, the cross-section of NbTi wire is not square; therefore, the cross-section of the NbTi filament and copper matrix is also represented by an ellipse to illustrate the actual geometry. The number of NbTi filaments in the microscale RVE was determined by the cross-sections of real samples and the discretization meshes, which are illustrated in a later section.

### 2.2. The Self-Consistent Analysis for the Microscale Model

At this point, the two-scale geometric model of the racetrack NbTi superconducting coil is established, but the solving methods have not been determined. Regarding the two-scale model, a direct and general method is utilizing the finite element method to solve the two models sequentially; for example, homogenization of the microscale model can be easily performed, and the equivalent parameters can be assigned in the macroscale models for further simulation. Although this is an effective method, it may be impossible to accurately describe the nonlinear behaviors of the microscale models, and the connection between the two models is unidirectional. Therefore, the microscale and macroscale models should communicate and exchange information during the solving procedures, which requires a bidirectional connection between the two models. The bidirectional method is more suitable for multiscale analysis, and the reduced-order method should be adopted in microscale models to reduce the significant computational resources required for multiscale analysis.

The self-consistent analysis (SCA) method is a recently developed reduced-order method that utilizes the concentration tensors of grid points and divides them into separate clusters. It iteratively solves the cluster-based Lippmann–Schwinger (L–S) equations to obtain local microscale responses. SCA partly inherits the framework of the classical fast Fourier transform (FFT) method [25,26] and incorporates the data-driven clustering technique, which avoids repetitive point-wise operations and maintains high computation efficiency. A brief introduction of SCA is presented below, which consists of offline and online stages, and interested readers can refer to it for more details.

In the offline stage, the main task is assigning the cluster region and calculating the corresponding interaction tensors. Firstly, the original geometry is discretized into formal grid points, and the K-means clustering algorithm is adopted to determine the similarities between the concentration tensors of grid points and separate them into clusters. The concentration tensor $A\left(x\right)$ can be obtained using the finite element method or FFT [27]:

(1) $$\varepsilon^{micro}\left(x\right)=A\left(x\right):\varepsilon^{macro}$$

where $\varepsilon^{micro}\left(x\right)$ and $\varepsilon^{macro}$ are the local strain at the grid point and the applied homogeneous macroscopic strain. The symbol **:** represents the double inner product. $A\left(x\right)$ can be computed by applying six sequential orthogonal loadings in three dimensions. Then, the interaction tensor $D^{IJ}$ between the clusters *I* and *J* can be computed [28]:

(2) $$\begin{array}{l} D^{IJ}=\frac{1}{c^{I}\left|\Omega \right|}\iint_{\Omega }\chi^{I}\left(x\right)\chi^{J}\left(y\right)\Gamma^{0}\left(x-y\right)dydx\\ \chi^{I}\left(x\right)=\left\{\begin{array}{l} 1\;\;\;x\in \Omega^{I}\\ 0\;\;\;otherwise \end{array}\right. \end{array}$$

where $c^{I}$ and $\left|\Omega \right|$ are the volume fraction of cluster *I* and the total volume of the RVE, respectively. $\Gamma^{0}$ represents the Green function operator with an explicit expression in Fourier space. The components of $D^{IJ}$ only need to be computed once in the offline stage.

In the online stage, the cluster-based L–S equation must be solved iteratively:

(3) $$\Delta \varepsilon^{I}+\sum_{J=1}^{k}D^{IJ}:\left[\Delta \sigma^{J}-C^{0}:\Delta \varepsilon^{J}\right]-\Delta \varepsilon^{0}=0$$

where $\Delta \varepsilon^{I}$, $\Delta \sigma^{J}$, and $\Delta \varepsilon^{0}$ are the incremental strain, incremental stress, and applied incremental homogenized strain, respectively. Equation (3) relies on the value of isotropic reference stiffness $C^{0}$ for the solution. Therefore, the reference stiffness $C^{0}$ should be approximate to the effective tangential stiffness $\bar{C}$:

(4) $$\bar{C}=\sum_{I=1}^{k}c^{I}C_{alg}^{I}:A^{I}$$

where $C_{alg}^{I}$ is the algorithm stiffness of cluster *I*. To compute the reference stiffness $C^{0}$, a projection algorithm is adopted:

(5) $$\begin{array}{l} C^{0}=\left(J::\bar{C}\right)J+\frac{1}{5}\left(K::\bar{C}\right)K\\ J_{ijkl}=\frac{1}{3}\delta_{ij}\delta_{kl},K_{ijkl}=\frac{1}{2}\left(\delta_{ik}\delta_{jl}+\delta_{il}\delta_{jk}\right)-\frac{1}{3}\delta_{ij}\delta_{kl} \end{array}$$

According to the aforementioned calculation in the offline and online stages, the mechanical responses of each cluster can be obtained, and the homogenized stresses and strains can also be computed by a weighted sum with volume fraction.

### 2.3. The Multiscale Analysis Based on Self-Consistent Analysis

By combining the finite element method and self-consistent analysis (FEM-SCA), two-scale analysis can be conducted for the racetrack NbTi superconducting coil. The macroscale model is solved using FEM, where the symmetric boundary conditions are implemented and the external dynamic loads are applied. Since the NbTi wires are not fully fixed in the stainless-steel coating, the surface contact between the inner NbTi wires and the outer steel coating is considered. The contacts between the NbTi wires are also discussed in Section 3. At the same time, the microscale model is solved using SCA, where the macroscopic strains are applied in the RVE and the corresponding macroscopic stresses are obtained by solving L–S equations. The corresponding equations for the two-scale FEM-SCA method are as follows:

(6) $$\left\{ \begin{array}{l} \nabla \cdot \sigma^{1}\left(x^{1}\right)+b^{1}=0,\forall x^{1}\in \Omega^{micro}\\ \nabla \cdot \sigma^{2}\left(x^{2}\right)=0,\forall x^{2}\in \Omega^{micro}\\ \varepsilon^{n}\left(x^{n}\right)=\frac{1}{2}\left(\nabla u^{n}\left(x^{n}\right)+{\left(\nabla u^{n}\left(x^{n}\right)\right)}^{T}\right),n=1,2\\ \sigma^{n}\left(x^{n}\right)=\left\{ \begin{array}{c} \frac{1}{\left|\Omega^{n+1}\right|}\int_{\Omega^{n+1}}\sigma^{n+1}\left(x^{n+1}\right)d\Omega^{n+1},n=1\\ f^{n}\left(\varepsilon^{n}\right),n=2 \end{array}\right.\\ u^{1}\left(x^{1}\right)=u_{h},x^{1}\in \Gamma_{h},t^{1}\left(x^{1}\right)=\sigma^{1}\cdot n^{1}=t_{s},x^{1}\in \Gamma_{s}\\ u^{n}:\mathrm{periodic},t^{n}:\mathrm{anti-periodic},n=2 \end{array}\right.$$

where *n* = 1 and 2 represent macroscale and microscale, respectively. In this paper, the NbTi filament and stainless steel are regarded as isotropic materials, and the copper matrix is assumed to be an elastoplastic material with an isotropic hardening law. The yield function can be fitted and explicitly written as [18]:

(7) $$\sigma_{Y}\left(\bar{\varepsilon }\right)=0.0372+1.1887{\bar{\varepsilon }}^{0.7943}$$

where $\sigma_{Y}$ and $\bar{\varepsilon }$ are the yield stress (GPa) and the equivalent plastic strain. The material parameters were obtained from the literature [18,29] and are summarized in Table 1.

**Figure 3:**
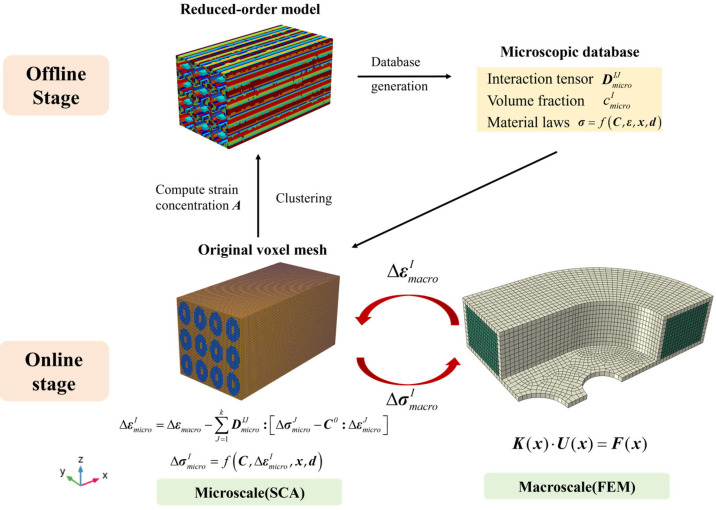
The concurrent FEM-SCA scheme for the racetrack NbTi superconducting coil.

In two-scale analysis, SCA is employed in the microscale RVE, and the FEM is implemented in the macroscale coil. Every macroscopic element of the NbTi wire array in the racetrack NbTi coil corresponds to an individual microscopic RVE. The data transfer between the two scales is bidirectional and concurrent. It should be noted that only the constitutive relationship of individual components—such as the NbTi filament, copper matrix, and stainless steel—needs to be defined. The overall mechanical responses of the NbTi wire arrays can be calculated as the weighted average of the microscale RVE. The detailed diagram of the FEM-SCA scheme for the racetrack NbTi superconducting coil is shown in Figure 3.

## 3. Numerical Validation of the Microscale Model

In the cross-section of the racetrack NbTi coil multiscale model, the coil contains 2304 wires, and every 12 wires form a microscopic model in the *y*-*z* plane, which means that the computational efficiency and accuracy of the multiscale simulation are relevant to the computation of the microscale model. Comparisons of the overall and local responses between the SCA and FEM methods were performed to assess the precision and computational cost. Meanwhile, an SCA convergence study of the microscale model was conducted to select the optimal cluster number, which should balance efficiency and accuracy.

### 3.1. The Overall Responses Comparisons

**Figure 4:**
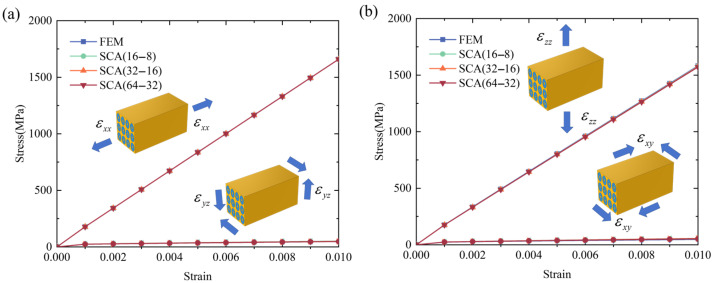
The stress–strain curves of the FEM and SCA microscale model under different loading conditions (**a**) uniaxial tension along the *x* axis and pure shear along the *y*-*z* plane; (**b**) uniaxial tension along the *z*-axis and pure shear along the *x*-*y* plane).

The original microscale model was divided into voxel meshes and separated into discrete clusters for SCA computation. The voxel mesh was 100 × 50 × 50, considering the geometry of the RVE, and the SCA clustering numbers were 16–8, 32–16, and 64–32, corresponding to the material region of the copper matrix and NbTi filament. The FEM model was also constructed for comparison, and the periodic boundary conditions were implemented. The different tension and shear loading conditions were applied in the SCA and FEM, and the stress–strain curves of the overall responses between these methods are shown in Figure 4. The results show that the SCA and FEM predictions were consistent for uniaxial loading and pure shear conditions, and the maximum discrepancies were within 5%. As for the comparisons between different cluster numbers, the stress–strain curves were close, which means that choosing a small cluster number can also obtain a high accuracy of overall responses.

**Figure 5:**
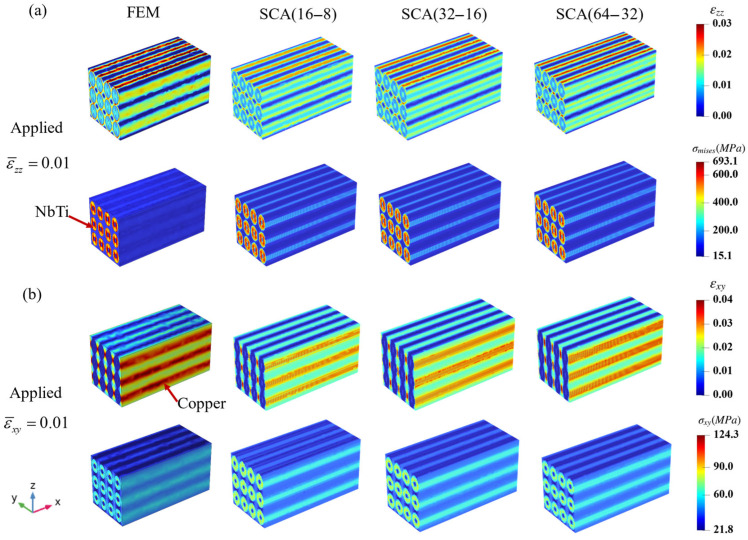
The overall strain and stress distribution results for FEM and SCA under different loading conditions (**a**): applied ${\bar{\varepsilon }}_{zz}=0.01$, (**b**): applied ${\bar{\varepsilon }}_{xy}=0.01$.

To further study the prediction of the overall strain and stress distribution within the RVE using the SCA method, the strain and stress distribution comparisons of the RVE using different methods are shown in Figure 5. Figure 5a shows the distribution results of $\varepsilon_{zz}$ and $\sigma_{mises}$ within the RVE under uniaxial tension loading according to different methods. It can be seen that the peak $\varepsilon_{zz}$ occurs in the copper matrix, while the peak $\sigma_{mises}$ occurs in the NbTi filaments. The reason may be that the copper underwent elastoplastic deformation, and the NbTi filaments bore the most stresses.

The distribution of $\varepsilon_{xy}$ and $\sigma_{xy}$ in Figure 5b also shows a similar pattern. We observed that the strain and stress distributions of the NbTi filaments and copper matrix depicted evident array-like characteristics, which meant the responses of a single strand were close to the responses of twelve strands under periodic loading conditions. When the number of clusters increased, the SCA results were closer to those of FEM, the distribution discontinuity was reduced, and the prediction of the peak region was more accurate.

### 3.2. The Local Response Comparisons

**Figure 6:**
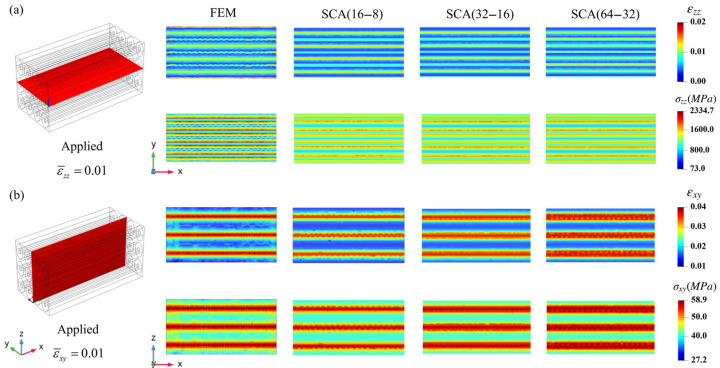
The local strain and stress distribution results for FEM and SCA at different cross-sections of the microscale RVE (**a**): applied ${\bar{\varepsilon }}_{zz}=0.01$, (**b**): applied ${\bar{\varepsilon }}_{xy}=0.01$.

To compare the local responses between the FEM and SCA results, the strain and stress distributions at the cross-section of the microscale RVE are shown in Figure 6. Figure 6a shows the $\varepsilon_{zz}$ and $\sigma_{zz}$ distributions at the *x*-*y* plane cross-section, where the strain and stress distributions of the NbTi filaments and copper matrix show a symmetric pattern. Considering the array-like geometry, the observed phenomenon is reasonable. The copper matrix shows higher strain values and NbTi filaments bear more stresses, which is consistent with Section 3.1. The discrepancies among different cluster numbers were not evident, and most regions showed similar distributions; only the peak region varied, possibly due to the discrete degree of different cluster numbers. Figure 6b shows the $\varepsilon_{xy}$ and $\sigma_{xy}$ distributions at the *x*-*z* plane cross-section, where the geometry only covers the copper matrix. The three peak strain regions, which are caused by the extrusion of adjacent NbTi filaments, can be clearly seen in the middle. Therefore, the peak $\sigma_{xy}$ stresses also occurred in the same regions. Although only 16 clusters were utilized in SCA (16–8) for copper matrix clustering, the peak and valley regions of the strains and stresses were in good agreement with FEM, which implies high SCA prediction precision with a small cluster number, and refined local precision with increased cluster number.

**Figure 7:**
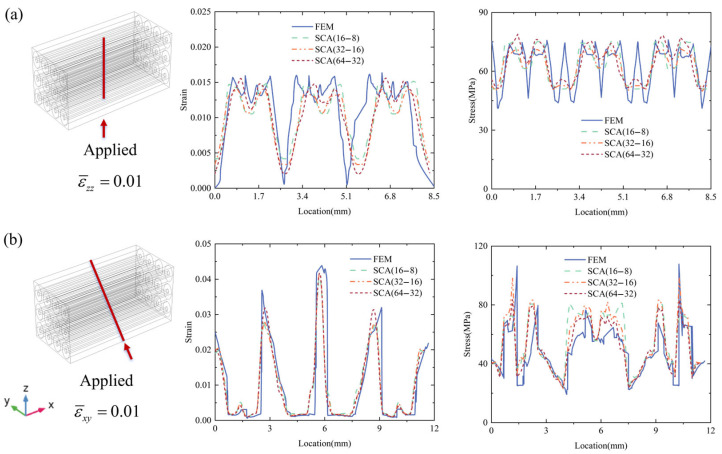
The local strain and stress distribution results for FEM and SCA along the red lines of the microscale RVE (**a**): applied ${\bar{\varepsilon }}_{zz}=0.01$, (**b**): applied ${\bar{\varepsilon }}_{xy}=0.01$.

The local strain and stress distributions along selected red lines are shown in Figure 7. Figure 7a depicts the $\varepsilon_{zz}$ and $\sigma_{mises}$ distributions along the red line at the middle of the microscale RVE, where the strain and stress show dramatic fluctuations due to the interaction of the NbTi filaments and copper matrix. The variation tendency between FEM and SCA is consistent, although differences exist between the local peak and valley values. Figure 7b depicts the $\varepsilon_{xy}$ and $\sigma_{xy}$ distributions along the red diagonal line of the microscale RVE, where several peak $\varepsilon_{xy}$ strain regions can be observed that correspond to the valley $\sigma_{xy}$ regions. The peak strain regions correspond to the copper matrix, and the other regions correspond to the NbTi filaments. The figures indicate that the cluster number has a negligible effect on segmentation precision. Notably, a small cluster number can achieve high precision for both uniaxial tension and pure shear loading conditions.

Finally, the time cost comparison between SCA and FEM is shown in Table 2 for a comprehensive analysis of the computational efficiency. The original grid number of the SCA method was 100 × 50 × 50 before it underwent clustering order reduction, while the FEM methods utilized 130,294 tetrahedron elements. It can be seen from the results in the table that the computing time of the offline stage of the SCA method increased with the number of clusters, while the computing time of the online stage changed slightly with the loading conditions. In the offline stage, only one calculation was needed to obtain the corresponding database. The same database could be utilized for different external loading conditions, and only the loading strains needed to be modified; therefore, the corresponding calculation results were quickly obtained by solving discrete L–S equations. As can be seen from the data in the table, even when the cluster number of SCA was 64–32, the computational efficiency in the online stage was nearly 860 times that of FEM. Considering the aforementioned results and calculation times for different cluster numbers, the cluster number of 32–16 achieved a good balance between accuracy and efficiency. Therefore, in the subsequent multiscale analysis, the number of clusters adopted in the SCA method was 32–16.

## 4. Two-Scale Analysis for the Racetrack NbTi Superconducting Coil

The racetrack NbTi superconducting coil can provide strong and homogeneous magnetic fields at the center, with wide applications for acceleration magnets and nuclear magnetic resonance devices [30]. Since the racetrack NbTi superconducting coil consists of many NbTi wires, contact and friction between the NbTi wires can pose potential challenges regarding the stable operation of the coil. Hence, the macroscopic contacts between different strands were investigated using the two-scale analysis for the racetrack NbTi superconducting coil, and the local responses of the microscopic filaments and copper matrix were also studied. In addition, considering the possible harmonic impact, the amplitude curve of the loading pressure was adjusted to satisfy the trigonometric function curve, and the responses under different loading times were also researched.

### 4.1. The Two-Scale Analysis Under Monotone Increasing Pressure

**Figure 8:**
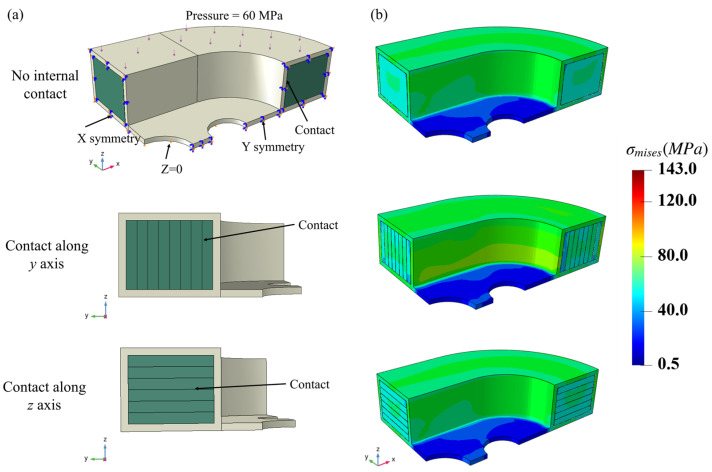
The two-scale model with different contact settings (**a**) and the corresponding macroscopic $\sigma_{mises}$ stress distributions (**b**).

The quarter racetrack NbTi superconducting coil consists of inner NbTi wire arrays and outer stainless steel coatings, where the inner region and outer region contain 9408 and 2788 C3D8R elements, respectively. Since the macroscopic coil model is a quarter, symmetric boundary conditions were applied at the edges of the macroscopic model. The pressure was applied to the top of the macroscopic model and increased monotonically in time steps up to a maximum of 60 MPa. As for the contact settings, we established three different models to study the effects of internal contacts between the strands: ‘No internal contact’ means only the contacts between the strands and the steel are studied, and no internal contacts between the strands are considered; ‘Contact along the *y* axis’ adds the contacts between the eight strand arrays along the *y* axis; and ‘Contact along the *z* axis’ adds the contacts between the six strand arrays along the *y* axis. The three models reflected the contact settings between the NbTi wire arrays shown in Figure 8a. Whole contacts used a surface-to-surface type with a penalty method, and the friction coefficient was 0.1.

Figure 8b shows the corresponding macroscopic $\sigma_{mises}$ stress distributions when the loading step is finished. Since the steel in the middle region was not subjected to external pressure, the $\sigma_{mises}$ stress value was low and the maximum stress value occurred at the bottom of the steel coatings. The stress distributions of the NbTi wire arrays and adjacent steel were approximate and uniform, which means the overall outer surfaces of the coil bore uniform loads. The results of different contact settings are not evident from the perspective view of the outer surfaces, which means the contacts between the NbTi wire array might have little effect on the overall $\sigma_{mises}$ stress.

**Figure 9:**
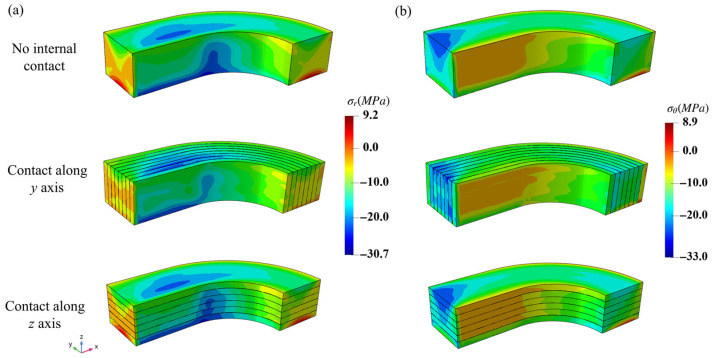
The macroscopic $\sigma_{r}$ (**a**) and $\sigma_{\theta }$ (**b**) stress distributions of the NbTi wire arrays under different contact settings.

Therefore, the cylindrical coordinate system was introduced to further study the responses of the NbTi wire arrays, where the radial axis is along the radius of the arc region and the azimuthal angle corresponds to the circumferential direction. The distributions of the $\sigma_{r}$ and $\sigma_{\theta }$ stresses of the NbTi wire arrays are shown in Figure 9. The overall distributions were similar between the three models, and the local stresses were more influenced by the different contact settings. Since the steel coating surrounds the NbTi wire arrays and bears most loads, the absolute maximum $\sigma_{r}$ and $\sigma_{\theta }$ stress values of the NbTi wire arrays were within 33 MPa. As the external loads were applied along the *z* axis, the distribution results of the ‘No internal contact’ and ‘Contact along the *z* axis’ settings were consistent; only the ‘Contact along the *y* axis’ setting had small differences at the edges. The distributions of $\sigma_{r}$ stress along the radial axis and $\sigma_{\theta }$ stress along the azimuthal angle showed irregular patterns due to the contact interaction and uniform pressure.

**Figure 10:**
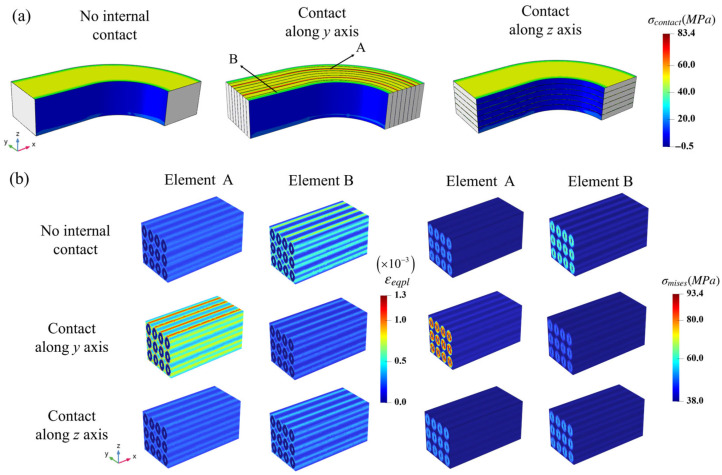
The macroscopic contact pressure (**a**) and corresponding microscopic $\varepsilon_{eqpl}$ and $\sigma_{mises}$ distributions for elements A and B (**b**) under different contact settings.

The contact pressure comparisons of the NbTi wire arrays are also shown in Figure 10a, which clearly indicates the internal contact pressure differences between the different contact settings. The ‘No internal contact’ and ‘Contact along the *z* axis’ settings produced high and homogeneous contact pressures at the top surface, while the setting ‘Contact along the *y* axis’ produced discrete contact pressures and generated peak value regions at the same position. This phenomenon indicates that the extrusion pressures between the NbTi array strands can be large. The maximum applied external pressure was 60 MPa; however, the peak contact pressure was 83.4 MPa, indicating that the effects of contact pressures between the NbTi wires should be considered in the coil design.

To study the microscopic responses related to the high and low contact pressure macroscopic regions, two macroscopic elements A and B were selected, and the distribution of the corresponding microscopic $\varepsilon_{eqpl}$ (equivalent plastic strain) and $\sigma_{mises}$ are shown in Figure 10b. The peak $\varepsilon_{eqpl}$ strain and $\sigma_{mises}$ stress appear in the microscopic result in the ‘Contact along the *y* axis’ setting, where the peak $\varepsilon_{eqpl}$ occurs in the top copper matrix region with a maximum value of $1{.3\;\times \;10}^{-3}$, and the peak $\sigma_{mises}$ occurs in the NbTi filaments with a maximum value of 93.4 MPa. Both the peak strain and stress values are almost twice as high as those for the other contact settings, indicating that the higher loads caused by contact pressures influence the mechanical response of microscale models. Furthermore, there exist minor differences between the microscopic results in the ‘No internal contact’ and ‘Contact along the *z* axis’ settings, which could be caused by the contact pressures between different layers along the *z* axis.

### 4.2. The Two-Scale Analysis Under Periodic Pressure

**Figure 11:**
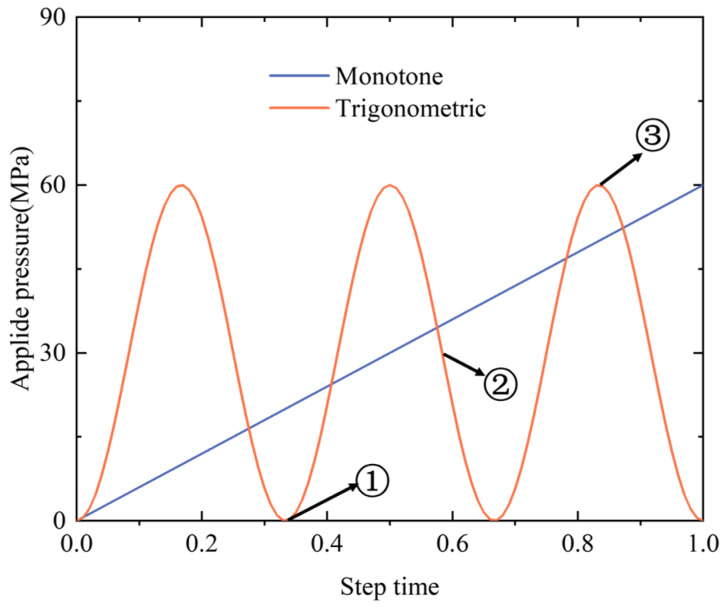
The applied pressure following a monotonic ramp and periodic trigonometric function.

For the racetrack NbTi coil used in Maglev trains, the harmonic impact could be an important loading condition; therefore, the loading pressure was adjusted to follow the periodic trigonometric function curve to simulate the periodic change of impacts in a real operation environment. The maximum value of pressure remained 60 MPa, whereas three full loading cycles replaced the monotonic ramp within the same timeframe, as shown in Figure 11. The indices j, k, and l represent the different loading times during the whole computation procedure.

**Figure 12:**
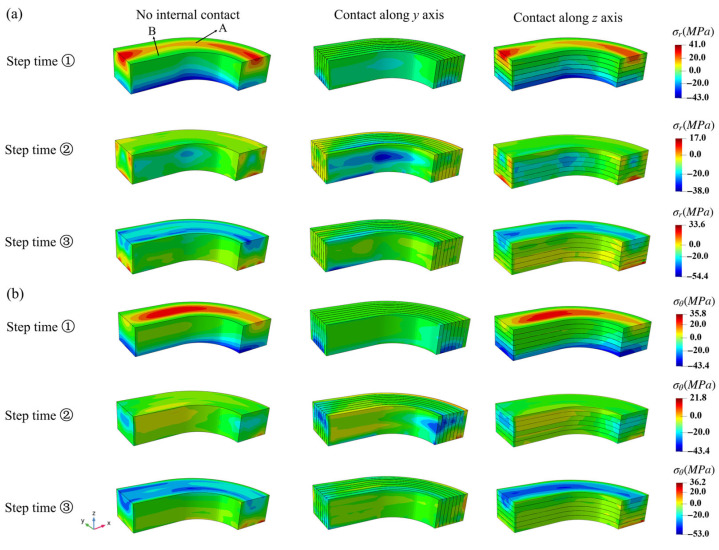
The overall $\sigma_{r}$ (**a**) and $\sigma_{\theta }$ (**b**) stress distributions of the NbTi wire arrays at different step times under periodic pressure.

The overall $\sigma_{r}$ and $\sigma_{\theta }$ stress distributions of the NbTi wire arrays at different step time under periodic pressure are shown in Figure 12. The absolute maximum values of $\sigma_{r}$ and $\sigma_{\theta }$ stress were 54.4 and 53 MPa, respectively, and both were negative, suggesting that most stresses along the radial and circumferential direction were compressive. From step time j to k to l, the positive $\sigma_{r}$ and $\sigma_{\theta }$ stresses gradually changed to negative values in most regions, and both stresses had similar varied tendency and values. The peak stress occurred at the top region, with a positive value at step time j, then it changed to a negative value at the middle and top region at step times k and l. The ‘Contact along *y* axis’ setting produced smaller peak stress values compared to the other contact settings.

**Figure 13:**
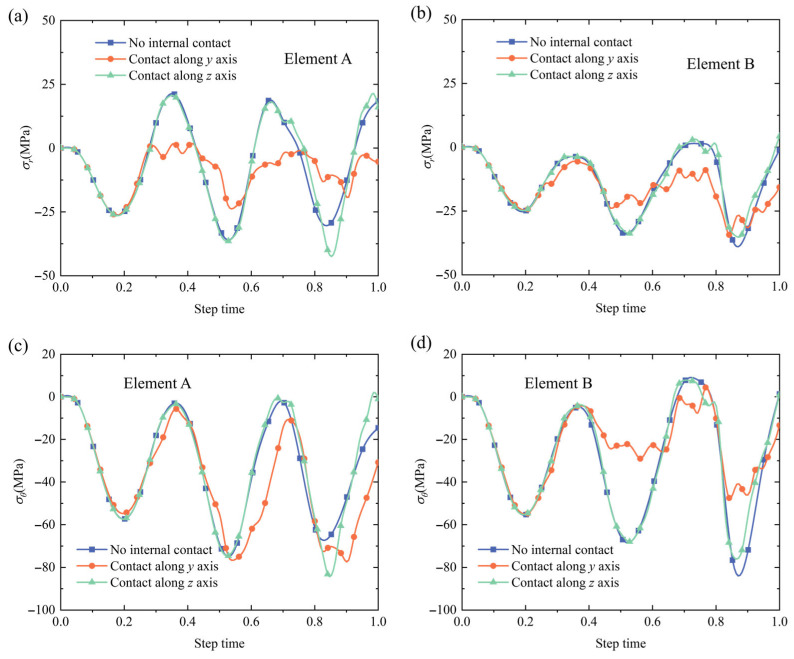
$\sigma_{r}$ (**a**,**b**) and $\sigma_{\theta }$ (**c**,**d**) stress variations for elements A and B under periodic pressure.

To illustrate the stress variations for the selected elements A and B in Figure 12, the $\sigma_{r}$ and $\sigma_{\theta }$ stress variations with full step times are shown in Figure 13. It can be observed that the stress variation trends were similar for elements A and B, and only the magnitudes showed small discrepancies. Compared to the loading pressure curve in Figure 11, the $\sigma_{r}$ and $\sigma_{\theta }$ stress variation tendencies for elements A and B were contrary to the loading pressure curve, indicating that the external pressures generated the internal compressive stresses between the NbTi wire arrays.

A notable phenomenon was that the absolute peak values of the $\sigma_{r}$ and $\sigma_{\theta }$ stresses increased with cycle times, and the maximum $\sigma_{r}$ and $\sigma_{\theta }$ stress values were approximately 40 and 80 MPa (both were negative), which occurred at a time step of 0.83. However, the maximum loading pressure was set at 60 MPa, and the increased $\sigma_{r}$ and $\sigma_{\theta }$ stresses could have occurred because the copper matrix underwent elastoplastic deformations and the NbTi filaments bore more loads as the cycle increased. The absolute stress value of the ‘Contact along the *y* axis’ setting was slightly smaller than that of the other contact settings, which means the stress values could be slightly smaller in actual service operation due to the contacts between the strands along the *y* axis.

**Figure 14:**
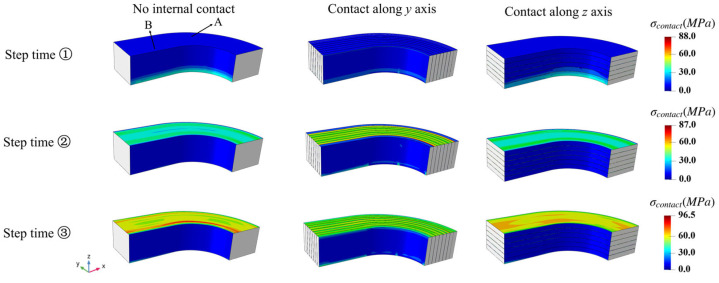
The contact pressures for the NbTi wire array at different step times under periodic pressure.

The contact pressures for the NbTi wire array at different step times are shown in Figure 14. This plot shows that the contact mainly occurred at the top and bottom regions of the NbTi wire arrays due to the applied pressure along the *z* axis, while the contact pressures along the circumferential direction were homogeneous. From step time j, k to l, the contact pressures at the top region increased owing to the rise in applied pressure loading.

**Figure 15:**
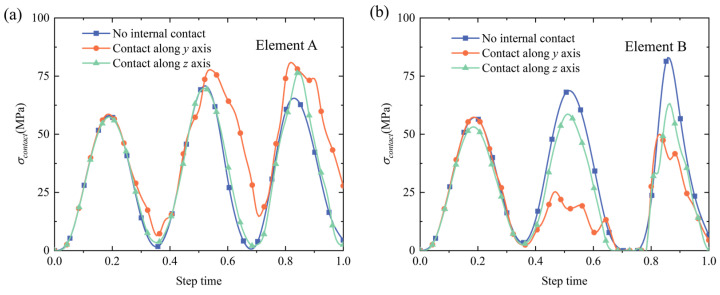
The contact pressure variations for elements A (**a**) and B (**b**) under periodic pressure.

The contact pressure variations for selected elements A and B are also shown in Figure 15. The contact pressures obeyed the periodic variation trend and increased with cycle time for element A. For element B variations, since the setting ‘Contact along the y axis’ considered the interactions between the NbTi wires along the y axis, the contact pressures for element B are more complicated, while the results of other contact settings followed the tendencies of element A. In summary, the peak contact pressure was more likely to appear at the middle region of the NbTi wire arrays under periodic pressure, which might require more attention when optimizing the design.

**Figure 16:**
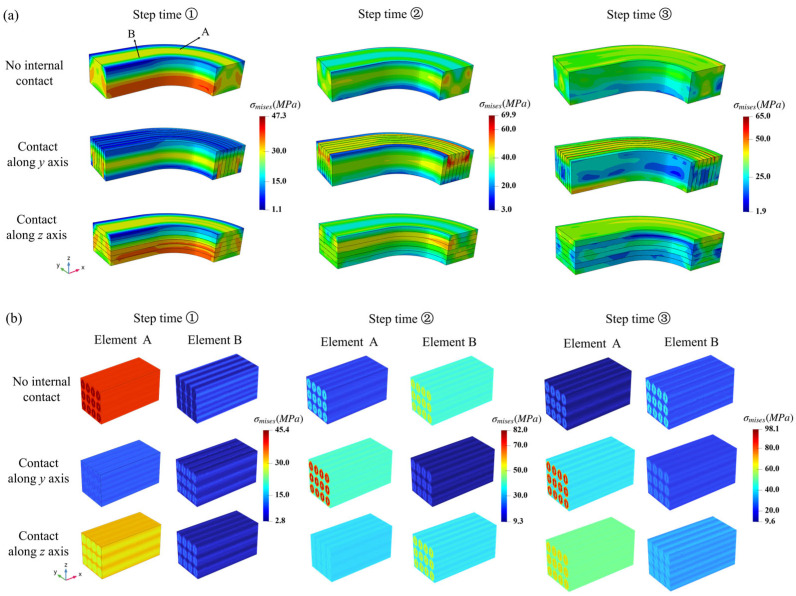
The macroscopic contact pressure (**a**) and corresponding microscopic $\varepsilon_{eqpl}$ and $\sigma_{mises}$ distributions for elements A and B (**b**) at different contact settings.

The macroscopic $\sigma_{mises}$ stress distributions of the NbTi wire arrays at different step times under periodic pressure are shown in Figure 16a, and the corresponding microscopic $\sigma_{mises}$ stress distributions appear in Figure 16b. Considering the overall stress variations, the magnitudes of $\sigma_{mises}$ stress increased with the applied pressure value. At step time k and l, the peak values occurred at the top region for the ‘Contact along the *y* axis’ setting. The result of the ‘Contact along the *z* axis’ setting illustrates the valley value at the middle layers along the *z* axis. Comparing the results of the internal contact settings with the results of the ‘No internal contact’ setting, the contact between the NbTi wires may have influenced the positions and values of peak and valley $\sigma_{mises}$ stress.

As for the microscopic responses, the $\sigma_{mises}$ stress magnitudes for element A were higher than the magnitudes for element B for most loading times. Element A was possibly more centered and subjected to greater effects from the interaction between the NbTi wires and upper steel coating, while element B was near the boundary and supported by the upper and lateral steel coating. Therefore, element B bore fewer loads than element A since most loads are distributed with lateral steel coating. Similar to the macroscopic $\sigma_{mises}$ variations, the magnitudes of microscopic $\sigma_{mises}$ stress followed the same tendency to increase with loading time. The peak values in the NbTi filaments had a maximum value of 98.1 MPa at time step l. It can also be seen that the maximum value of macroscopic stress was 65 MPa at time step l, and the maximum microscopic stress was higher than the macroscopic stress. We noted that the macroscopic responses were obtained according to the averaged values of the microscopic responses; therefore, the real peak value appeared in the microscopic model.

Finally, the computational time cost comparison between the FEM-SCA and FEM^2^ methods (both scales were computed using FEM, its calculation time was estimated) is summarized in Table 3. The FEM-SCA scheme only needed to generate the database once at the offline stage and directly used it in the multiscale analysis. However, the FEM^2^ scheme contained a large number of degrees of freedom and had a high computational cost. The presented FEM-SCA analysis demonstrated efficiency advantages, working almost 2900 times faster than the FEM^2^ analysis.

## 5. Conclusions

This study investigated the multiscale mechanical response of a NbTi superconducting coil under dynamic pressures using the FEM-SCA multiscale method. Since the NbTi coil consists of thousands of NbTi filaments, a macroscale coil model and a microscale RVE model were established, allowing both the macroscopic $\varepsilon_{eqpl}$ and $\sigma_{mises}$ stresses and contact pressures and the microscopic $\sigma_{mises}$ stresses and $\varepsilon_{eqpl}$ strains to be captured. The comparison results of the microscale models using SCA and FEM validated the high accuracy and efficiency of the established reduced-order model. The macroscopic responses at different contact settings under different applied pressures were discussed, suggesting that the peak contact pressure more likely appears at the middle region of the NbTi wire arrays under monotone and periodic pressure, and the internal contacts between strands can influence the distribution of stresses and contact pressures. Moreover, most regions in the macroscopic model showed increases in the magnitude of stresses and contact pressures with increasing cycle time, which means that fatigue and damage phenomena can occur with high cycle times. Finally, the microscopic stress distributions correspond to the macroscopic stress distribution, where the peak microscopic stress value is higher than the macroscopic stress value, suggesting that further failure behavior of the NbTi filaments can happen in the microscale model but may not be reflected in the macroscopic model. This phenomenon demonstrates how multiscale analysis can clarify the critical mechanical responses of the NbTi coil, which is impossible when performing single-scale analysis. The presented FEM-SCA framework is also combined with nonlinear damage evolution and coupled with electromagnetic field analysis, allowing us to understand and determine the multi-field responses of the racetrack NbTi superconducting coil in future work.

## Figures and Tables

**Table 1:** The elastic mechanical parameters of different materials [18,29].

Material	*E* (GPa)	*v*	*ρ* (kg/m^3^)
NbTi filament	111.7	0.36	6535
Copper matrix	139.6	0.34	8920
Stainless steel	210	0.28	7850

**Table 2:** The calculation time(s) for FEM and SCA with different cluster numbers (different direction loading times are averaged).

	Offline	Uniaxial Tension	Pure Shearing
		Online (Averaged)	Online (Averaged)
FEM (130,294 elements)	/	508	654
SCA(16–8)	7.07	0.18	0.14
SCA(32–16)	11.42	0.24	0.20
SCA(64–32)	57.75	0.59	0.69

**Table 3:** Comparison of computational times for the FEM-SCA and FEM^2^ methods.

	Elements/Clusters	DOFs	Computational Time	
			Offline	Online
FEM2	1.23 × 109	7.35 × 109	6.09 × 104 h (estimated)	
FEM-SCA	4.54 × 105	2.73 × 106	11.42 s	20.95 h

## Data Availability

The original contributions presented in this study are included in the article. Further inquiries can be directed to the corresponding author.

## Nomenclature

A	strain concentration tensor
b	body force
$C^{0}$	homogeneous reference stiffness
$C_{a\mathrm{lg}}^{I}$	algorithm stiffness tensor of the Ith cluster domain
$\bar{C}$	averaged effective tangential stiffness
$c^{I}$	volume fraction of the Ith cluster domain
$D^{IJ}$	cluster-wise interaction tensors
*E*	Young’s modulus
u	local displacement
$u_{h}$	the prescribed displacements on the boundaries
$t_{s}$	the prescribed traction forces on the boundaries
$\Gamma^{0}$	Green’s function
$\Delta \varepsilon^{0}$	applied macroscopic incremental strain
$\Delta \varepsilon^{I}$	incremental strain of the Ith cluster domain
$\Delta \sigma^{J}$	incremental stress of the Ith cluster domain
ε	local total strain
$\varepsilon^{\mathrm{micro}}$	local strain in the offline stage
$\varepsilon^{\mathrm{macro}}$	macroscopic strain in the offline stage
v	Poisson’s ratio
σ	local stress
$\sigma_{Y}$	yield stress
$\chi^{I}$	characteristic function

## Abbreviations

DOF	Degree of freedom
FEM	Finite element method
FFT	Fast Fourier transform
L–S	Lippmann–Schwinger
RVE	Representative volume element
SCA	Self-consistent clustering analysis
